# The glass walls of Samarra (Iraq): Ninth-century Abbasid glass production and imports

**DOI:** 10.1371/journal.pone.0201749

**Published:** 2018-08-22

**Authors:** Nadine Schibille, Andrew Meek, Mark T. Wypyski, Jens Kröger, Mariam Rosser-Owen, Rosalind Wade Haddon

**Affiliations:** 1 IRAMAT-CEB, UMR5060, CNRS / Université d’Orléans, Orléans, France; 2 Department of Scientific Research, The British Museum, London, United Kingdom; 3 Department of Scientific Research, The Metropolitan Museum of Art, New York, NY, United States of America; 4 Museum für Islamische Kunst, Staatliche Museen zu Berlin, Berlin, Germany; 5 Middle Eastern Section, Victoria and Albert Museum, London, United Kingdom; 6 Independent Scholar, London, United Kingdom; Seoul National University College of Medicine, REPUBLIC OF KOREA

## Abstract

Capital of the Abbasid Caliphate between 836 and 892 CE, the palace-city of Samarra offers a precise window into early Islamic art and architecture. Excavations conducted more than 100 years ago are seen as the beginnings of scientific Islamic archaeology, and have yielded an exceptional array of finds including a wealth of glass artefacts. The chemical composition of glass reflects the nature of the raw materials and their geological provenance and can therefore reveal past technologies and economic and cultural interactions. Through high-resolution analysis of a comprehensive glass assemblage from Samarra we have new evidence that points to the existence of an advanced Abbasid glass industry, as well as the import of specific glass objects for the thriving new capital city. Quantitative analytical data of 58 elements by laser ablation inductively coupled plasma mass spectrometry (LA-ICP-MS) show a striking correlation between object types and glass compositions. The compositional profiles of two related plant ash groups of architectural glass point to a local production, destined for the decoration of the famed glass walls of Abbasid palaces. The selective use of objects, materials and colours to create reflective and luminous glass walls are indicative of the great cultural and economic value of glass during the Abbasid period. Our findings thus confirm the veracity of written sources that stipulate the production of glass in the vicinity of Samarra, as well as the import of selected artefacts such as Byzantine mosaic tesserae.

## Introduction

The Arab conquest of Egypt and Syria-Palestine in the two decades following Muhammad’s death in 632 CE does not appear to have had an immediate impact on the primary production of glass in that region. Traditional large-scale Roman-type mineral soda based glassmaking continued well into the early Islamic period [1–4]. New raw glass compositions reflecting the use of soda-rich plant ash instead of mineral soda (*natron*) emerged only towards the end of the eighth and early ninth century both in Egypt and the Levant [2, 5–7]. The reasons underlying these changes remain unknown. However, they coincided with an increasing cultural eastward shift that began during the later Umayyad period and accelerated with the rise of the Abbasids and the foundation of Baghdad as the new capital in 762 CE [4, 8]. While nothing remains of the round city of Baghdad, the city of Samarra some 125 kilometres north of Baghdad preserves the art and archaeology of the early Abbasid period [9]. Samarra was founded in 836 CE by the eighth Abbasid caliph al-Mu‘tasim (r. 833–842) on the eastern bank of the Tigris as a vast palatial complex known as Dār al-Khilāfa [10]. The city underwent further development and expansion under his successors, most notably his nephew al-Mutawakkil (r. 847–861), who commissioned the Great Mosque of Samarra and additional palaces such as al-Mutawakkiliyya and Balkuwārā [11]. Samarra served as the administrative centre of the Abbasid Empire until 892 CE, when the Abbasid court returned to Baghdad, marking the end of Samarra’s caliphal period [9].

Archaeological excavations conducted at Samarra by a German expedition under Ernst Herzfeld in 1911 and 1912–13 revealed extensive architectural ornamentations of the palaces, including large numbers of glass artefacts [12]. Circumstantial evidence suggests that glass might have been worked or even made in Samarra. Al-Ya’qubi reports in his *Kitab al-Buldan*, the principal contemporary description of the foundation of Samarra, that the caliph al-Mu‘tasim ‘brought from al-Basra people who make glass’ [9], while nearby al-Qadisiyya was known in the thirteenth-century as the *glassworks* (ma‘mal al-zujaj) and as ‘the large village … where glass is made’ [9]. Site surveys of the area have yielded substantial debris of a possible glass industry, and ceramic finds testify to a continuous occupation of al-Qadisiyya from the Sasanian period through to the thirteenth or fourteenth century [9]. Another piece of evidence comes from the glass assemblage itself. Glass was put to innovative uses at Samarra in a wide range of architectural ornamentation such as mosaics, hollow diamond shaped, triangular, round or oval inlays of colourless transparent glass and shiny purple or millefiori tiles. The throne room of al-Mu‘tasim’s Dār al-Khilāfa ranks among the earliest and most important examples of architectural glass decorations (836–842 CE) [13], and written sources indicate that the lavish glass walls were symbolically charged [9, 14]. The proliferation of decorative architectural glass is one of the most distinguishing features in the archaeological record of Samarra and this type of glass is often supposed to have been made locally [12, 13].

To reconstruct the networks of supply and exchange, we conducted high-resolution analysis of major, minor and trace element compositions of a statistically significant number of well-dated glass samples from Samarra by LA-ICP-MS. These new compositional data are discussed in relation to published early Islamic glass assemblages, and identified both the selective import of specific object types as well as the regional production of glass at Samarra. Our findings bring about a radical reinterpretation of the scale and sophistication of production and interregional trade of glass during the ninth century. The evidence is furthermore concurrent with early Islamic textual sources. This is proof of the accuracy of these accounts that express a cultural identity and the importance of glass in early Islamic societies. The present work therefore considerably expands earlier analytical studies of Islamic glass assemblages [15–18] by relating the compositional data to artefact type and optical properties to assess the cultural and economic value of vitreous materials during the early Islamic period.

## Materials and methods

### Glass samples

**Fig 1:**
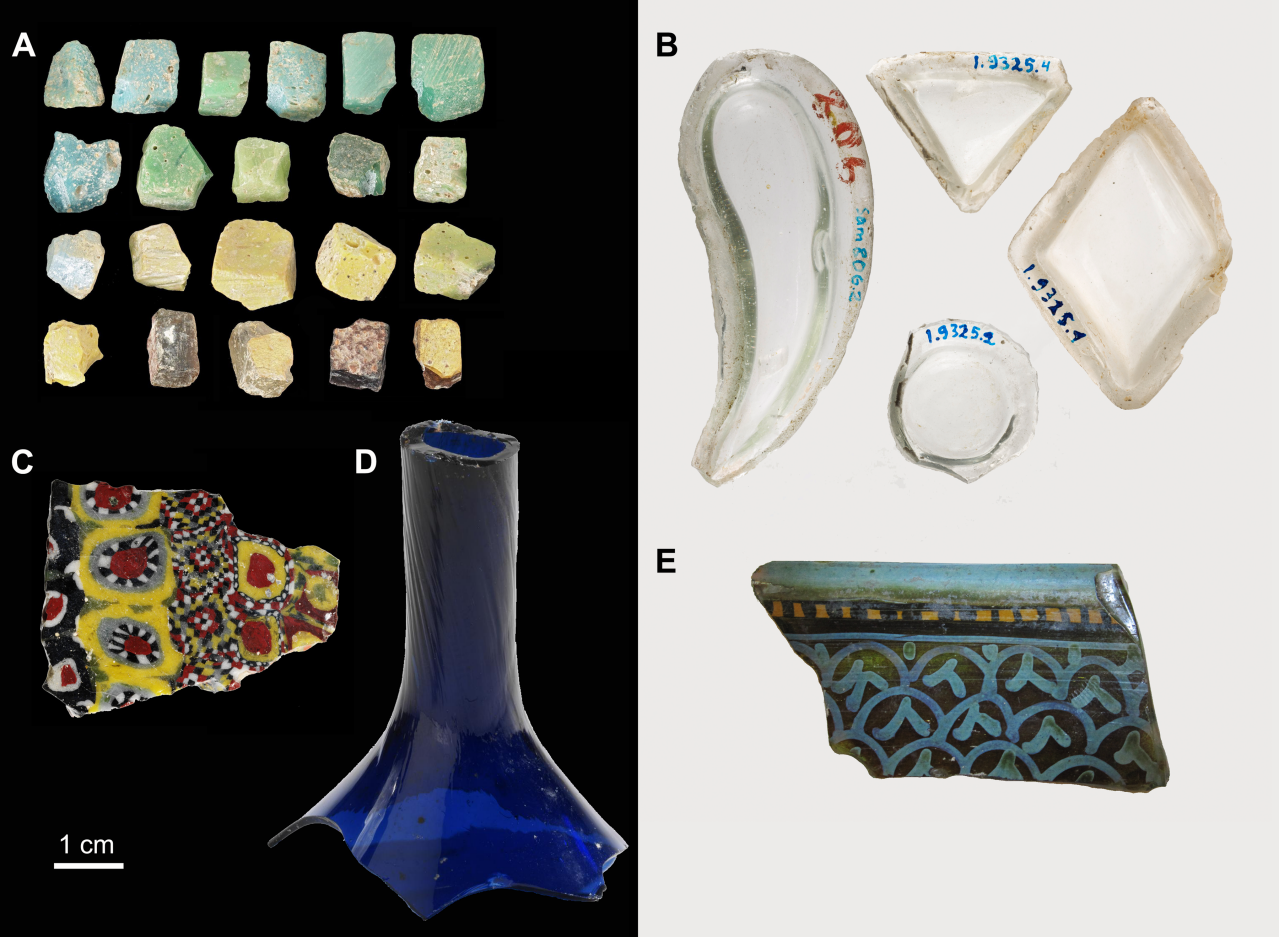
Glass artefacts from Samarra representing the different compositional groups. (**A**) Regularly shaped mosaic tesserae of natron type base glass (V&A A.58-1922); (**B**) glass inlays of plant ash group 1 (Sam 806.2, I. 9325.1, I. 9325.2, I. 9325.4; photos C. Krug, Museum für islamische Kunst / Staatliche Museen zu Berlin); (**C**) fragment of millefiori glass tile of plant ash group 2 (V&A C.743-1922); (**D**) cobalt blue flask neck (V&A C.750-1922); (**E**) rim fragment of painted glass bowl belonging to the miscellaneous samples (SamKat 273; photo M. Wypyski, Museum für islamische Kunst / Staatliche Museen zu Berlin). Images A, C and D from the Victoria and Albert Museum, London [http://collections.vam.ac.uk]; images B and E from the Museum für islamische Kunst / Staatliche Museen zu Berlin [www.smb-digital.de/eMuseumPlus].

The glass finds from Samarra have been published as part of the excavation reports in 1928 by Carl Johan Lamm [12], representing the first extensive publication on Islamic glass and Islamic archaeology more generally [19]. Among almost 400 catalogued glass finds now housed in various museum across the world are a large number of relatively simple blown and undecorated vessels, including bowls/plates, bottles, jugs and lamps, and mould blown vessels mostly of weakly coloured or cobalt blue glass, as well as more sophisticated vessels with pinched, stamped, engraved or linear wheel-cut decorations and a few examples with applied and painted surface decorations [12]. Numerous architectural glasses make up a large part of the assemblage, and include windows, monochrome or millefiori tiles, mosaic tesserae, drawn tesserae as well as hollow inlays of colourless transparent glass that are diamond shaped, triangular, round or oval. For the present study, 265 of the Samarra glass finds from the public collections of the Museum für islamische Kunst in Berlin (Germany), the Department of the Middle East in the British Museum and the Middle Eastern Section in the Victoria and Albert Museum in London (UK) were selected for analysis. No permits were required for the described study, which complied with all relevant regulations. The samples were chosen so as to include all vessel types (bottles, bowls, plates), optical properties (transparent, opaque, different colours) and decorative techniques (relief cut, engraved, painted, mould blown), and include all the architectural glasses (Fig 1 and S1 Table). Most of the glasses for which the archaeological context is known were either found in one of the palaces (Dār al-Khilāfa, Balkuwara) or in the Great Mosque, and the original catalogue numbers from Lamm are cross-referenced where possible (S1 Table).

### Analytical methods

Small fragments of glass were removed from the selected artefacts, set in epoxy resin and polished to remove any surface contamination. Laser ablation inductively coupled plasma mass spectrometry (LA-ICP-MS) analyses were performed on the polished samples at IRAMAT-CEB in Orléans (France), using a Thermofisher Element XR mass spectrometer and a Resonetic UV laser microprobe equipped with a 193 nm Excimer laser [20–22]. The laser was set at a 100 μm spot size that was occasionally reduced when manganese saturation occurred and analyses were carried out at 5 mJ with a frequency of 10 Hz, a pre-ablation time of 20 seconds followed by 30 seconds analytical time. Quantitative wt% and ppm concentrations of the fifty-eight elements measured were calculated, using ^28^Si as internal standard and a range of well-characterised glass reference materials (Nist SRM610, Corning B, C and D, APL1). To establish precision and accuracy, glass standards NIST SRM612 and Corning A were run at regular intervals (S2 Table). The detection limits vary between 0.1 and 0.01% for major elements and between 20 and 500 ppb for trace elements.

## Results

### Glass group provenance

The chemical fingerprints obtained by LA-ICP-MS classify all samples from Samarra as soda-lime-silica glasses typical of late Byzantine and early Islamic assemblages from the eastern Mediterranean and Mesopotamia (S1 Table). The majority of the samples have relatively high levels of potash (K_2_O > 1.5%) and magnesia (MgO > 2%), indicating that a soda-rich plant-ash served as the source of the alkali [23]. A small group of samples has low potassium and magnesium concentrations (< 1.5%) characteristic of glass made with soda derived from a mineral source. They are natron-type glasses characteristic of glass produced prior to the ninth century CE. The emerald green decoration of one vessel fragment (Berlin Sam 014) with approximately 70% lead oxide corresponds to a high lead silica glass (S1 Table). Similar compositions have been identified among early Islamic glasses, for example, from the Serçi Limani shipwreck [24] as well as Nishapur [25]. This sample will not be discussed further.

**Fig 2:**
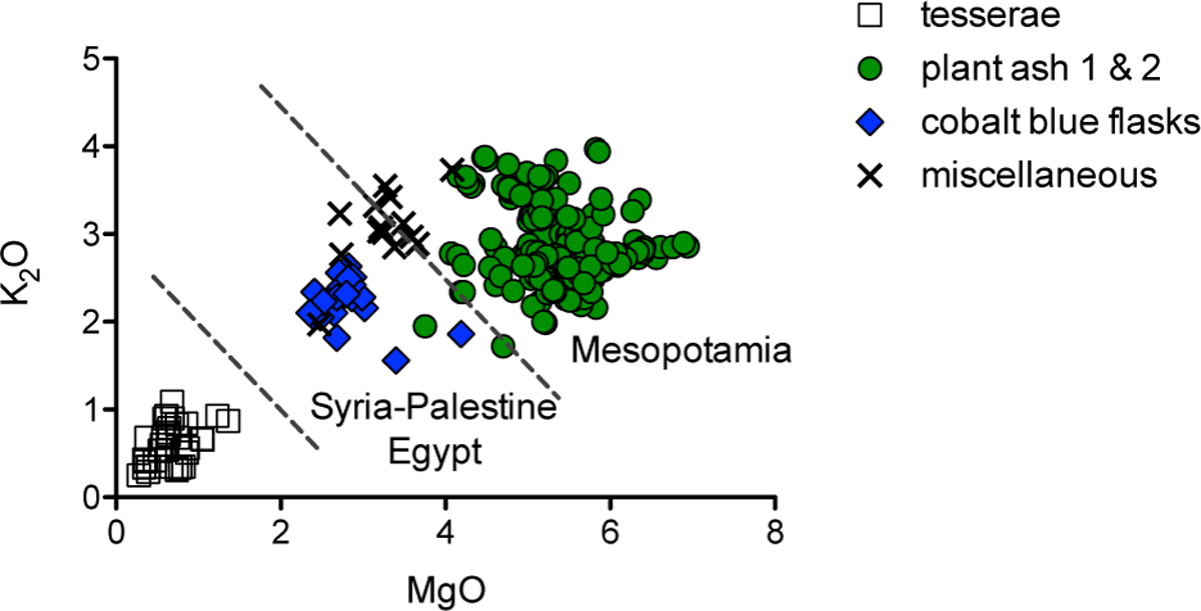
Different fluxing agents of the Samarra glasses. K_2_O versus MgO concentrations identify differences between natron-type glasses, plant ash glasses from the Syro-Palestinian Islamic tradition and plant ash glasses of Mesopotamian provenance (sub-divisions are indicated by dashed lines).

The analyses identified three different sources of supply: the reuse of older natron-type glass, imports of contemporary plant ash glass from the Levant and/or Egypt, and Mesopotamian glass production (Table 1 and Fig 2). Remarkably, the analytical results show a close correspondence between the compositional groups and object types. Almost all traditional regularly shaped tesserae (Fig 1A) and one colourless beaker with linear wheel cut decorations (Berlin Sam 038 MW) were produced from natron-type glasses sourced from the Levantine coast or Egypt. The plant ash glasses can be further subdivided into Mediterranean and Mesopotamian glass groups (Fig 2) that correspond roughly with artefact types and/or colours and by extension the quality of the glass. The colourless diamond shaped and round wall inlays as well as some delicately decorated vessels are made of a particularly high quality glass of Mesopotamian origin, while the strongly coloured architectural glasses and the majority of aqua coloured vessels derived from a silica source of Mesopotamian provenance that was of a lower purity. A third plant-ash group (henceforth referred to as miscellaneous) is relatively heterogeneous both in terms of its compositional traits as well as the typology and decorative techniques employed, suggesting the import of individual objects to Samarra (S1 Table). Finally, a chemically relatively tight cluster of cobalt blue flasks exhibits clear Mediterranean characteristics. With each compositional group being represented by at least 15 samples (> 5% of the assemblage), the relative standard errors of the mean (RSEM) are generally ≤ 5% for all base glass elements (Table 1). This shows that our group assignment is robust, which allows us to draw statistically and archaeologically significant conclusions from our data.

**Table 1:** Means and relative standard error of the mean (RSEM) of the five glass groups identified at Samarra. Data [wt %] were reduced to the shown major, minor and trace element oxides and normalised to 100%.

		Na_2_O	MgO	Al_2_O_3_	SiO_2_	P_2_O_5_	Cl	K_2_O	CaO	TiO_2_	MnO	Fe_2_O_3_	Sr [ppm]	Zr [ppm]
**Natron glass**	tesserae (n = 33)	16.9	0.72	2.72	69.1	0.15	1.02	0.65	7.00	0.14	0.57	1.04	411	72.9
	RSEM [%]	2.10	6.20	3.47	0.59	11.7	3.98	6.67	6.35	8.55	22.7	16.8	7.35	8.90
**Mesopotamian**	group 1 (n = 72)	13.2	5.09	0.81	70.0	0.12	0.64	3.22	6.39	0.03	0.29	0.23	398	31.0
	RSEM [%]	0.58	0.93	2.10	0.17	1.91	1.48	1.76	0.83	3.48	3.78	4.03	0.93	3.58
	group 2 (n = 136)	15.2	5.55	1.37	67.0	0.12	0.61	2.71	5.43	0.08	1.28	0.65	438	106
	RSEM [%]	0.73	0.85	1.08	0.25	1.75	0.78	0.87	0.94	1.37	4.13	4.63	0.86	1.87
**Imports**	Co flasks (n = 23)	14.3	2.83	2.10	67.6	0.19	0.71	2.26	6.66	0.13	0.59	2.56	332	95.7
	RSEM [%]	0.81	2.83	2.63	0.40	3.59	2.98	2.42	2.28	2.46	10.4	4.25	1.55	4.56
	misc (n = 15)	14.4	3.25	2.60	65.9	0.31	0.61	3.07	7.13	0.15	1.48	1.06	453	93.5
	RSEM [%]	3.25	3.17	13.7	1.48	4.49	5.34	3.38	5.88	11.3	21.1	13.1	7.92	10.9

### Reused tesserae of Levantine and Egyptian origins

**Fig 3:**
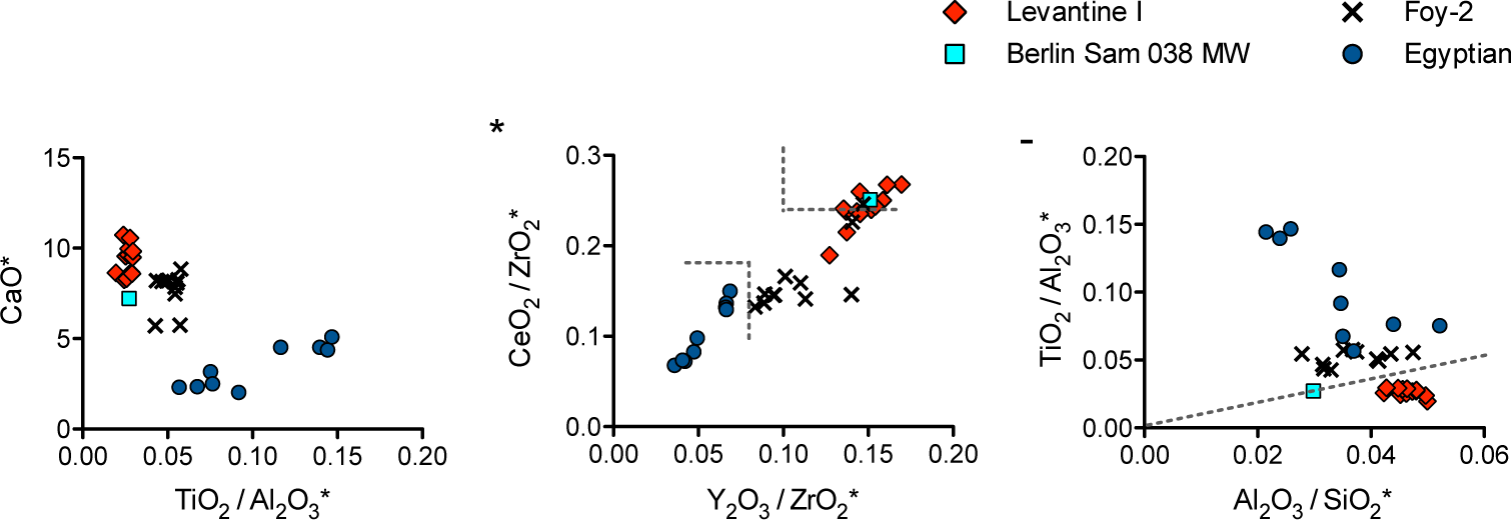
Analysis of the silica sources of the natron-type glasses from Samarra. (**A**) CaO versus the ratio of TiO_2_ / Al_2_O_3_; (**B**) CeO_2_/ZrO_2_ versus Y_2_O_3_/ZrO_2_; (**C**) TiO_2_/Al_2_O_3_ versus Al_2_O_3_/SiO_2_.

Upon closer inspection, the natron-type mosaic tesserae do not form a homogeneous group but show significant variations in their aluminium, calcium and heavy element concentrations reflecting different silica sources rather than secondary additives and thus different origins (Fig 3). About one third of the tesserae are characterised by moderate soda levels, alumina contents of about 3%, lime between 8% and 10.5% and low heavy mineral impurities consistent with Levantine I glass (Fig 3A). This type of glass has been identified among fourth- to eighth-century glass assemblages in Israel (Apollonia, Bet Shean, Dor and Jalame) [2, 26, 27]. A second group exhibits signs of recycling. It resembles so-called Foy-2 [28] on account of elevated heavy elements, relatively high soda levels and higher strontium to calcium ratios (S1 Table). Foy-2 is widespread among sixth- and seventh-century CE glass assemblages from diverse Mediterranean and European sites [22, 29–31]. The remaining samples have substantially lower calcium levels, high titanium, zirconium and hafnium, and varying titanium to aluminium ratios (Fig 3A). These features point to an Egyptian origin. The sub-group with the lowest lime and highest alumina concentrations resemble the characteristics of Egypt I, a primary production group dating to the eighth century CE based on a study of Islamic glass weights [32]. A single vessel fragment with low aluminium, titanium and relatively high calcium oxide concentrations and notable traces of manganese and antimony corresponds to recycled Roman glass [33].

Correlations between the geographical origin of natron-type glasses and the ratios of yttrium to zirconium versus cerium to zirconium, and titanium to aluminium versus aluminium to silica have been observed elsewhere [6, 31]. Glasses from Egypt have typically lower Y_2_O_3_/ZrO_2_ and CeO_2_/ ZrO_2_ ratios (< 0.08 and < 0.18, respectively) compared to glasses from the Levant (> 0.1 and > 0.24). Due to the higher alumina levels relative to the silica and titanium contents, Levantine I has simultaneously higher Al_2_O_3_/SiO_2_ (> 0.04) and lower TiO_2_/Al_2_O_3_ ratios (< 0.04) than Egyptian glass groups. Using these models, the initial assignments regarding the provenance of the Samarra tesserae are reinforced (Fig 3B and 3C). The tesserae that had been attributed to the Levantine I type have high Y_2_O_3_/ZrO_2_ and CeO_2_/ ZrO_2_ ratios as well as high alumina relative to silica and low titanium to alumina ratios (Fig 3B and 3C). The Egyptian groups have inverse characteristics. The provenance of Foy-2 is less clear, as the samples occupy an intermediate position as regards their Y_2_O_3_/ZrO_2_ and CeO_2_/ ZrO_2_ ratios that suggests some degree of mixing and/or recycling [6]. Recycling is confirmed by the elevated phosphorus and antimony contents of these samples (S1 Table).

The collection of mosaic tesserae retrieved from Samarra is evidently an eclectic mixture of different natron-type base glasses, all of which pre-date the foundation of Samarra in the ninth century. The tesserae were clearly not produced to a single recipe or a single commission. They might instead have been scavenged from buildings no longer in use and imported to Samarra from the western regions of the Abbasid Caliphate, from Syria-Palestine and/or Egypt. What all the tesserae have in common though is the fact that they are compositionally distinct from the output of the glassmaking industry that supplied Samarra with the bulk of soda-rich plant ash glasses.

### Regional production of plant ash glasses and imports

**Fig 4:**
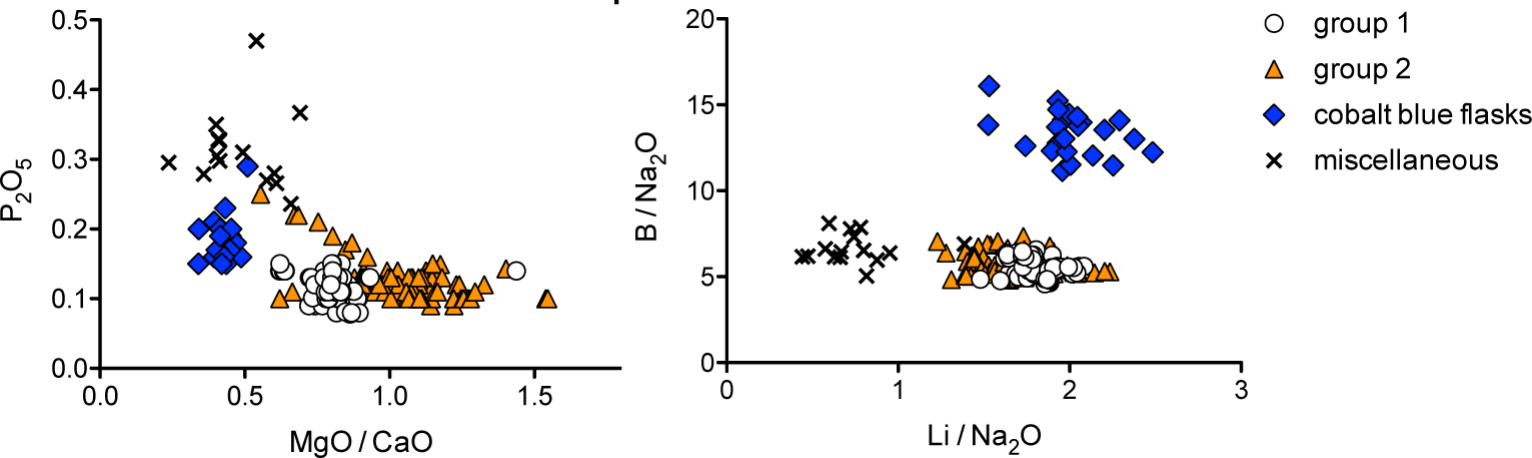
Variations in the plant ash component of the four plant ash groups. (**A**) phosphorus compared to magnesium to calcium oxide ratios confirm different geographical origins; (**B**) boron and lithium concentrations (both normalised to the soda concentrations) identify different plant ash components and additives.

Earlier studies have shown that Islamic soda-rich plant ash glasses from the Levant and Egypt can be distinguished from Mesopotamian plant ash glasses based on the potash and magnesia concentrations [34]. Applying a threshold of 6.5% for the sum of magnesium plus potassium oxide, the assemblage from Samarra comprises predominantly Mesopotamian plant ash glasses, whereas the cobalt blue flasks appear to represent a Syro-Palestinian or Egyptian production (Fig 2). The miscellaneous plant ash group has diverse features and consists of a mix of Mediterranean and Mesopotamian samples. The glasses of Mesopotamian group 1 and 2 are furthermore characterised by higher magnesium to calcium ratios and exceptionally low phosphorus contents (P_2_O_5_ < 0.15%) compared to the other plant ash types (Fig 4A). The lithium and boron levels relative to the soda concentrations separate the plant ash groups even more clearly (Fig 4B). The cobalt blue flasks form a neat cluster with higher boron levels than any of the other plant ash groups, while the miscellaneous plant ash group has the lowest lithium values relative to soda, which may be related to its overall lower magnesium contents (Fig 2). Groups 1 and 2 resemble each other closely in terms of most ash-related elements such as potassium, magnesium, lithium and boron but differ in their absolute calcium contents. Group 1 has on average higher calcium oxide levels (≈ 6.4%) compared to group 2 (≈ 5.4%) and accordingly somewhat lower magnesia to lime ratios (Table 1 and Fig 4A). These compositional features indicate differences in the plants that were used and/or in the preparation of the ash. Soda-rich plant ash was combined either with quartz-rich sand or with crushed quartz pebbles, providing additional elements to separate the groups and trace their likely origins.

#### High quality architectural glass—the use of quartz pebbles (group 1)

**Fig 5:**
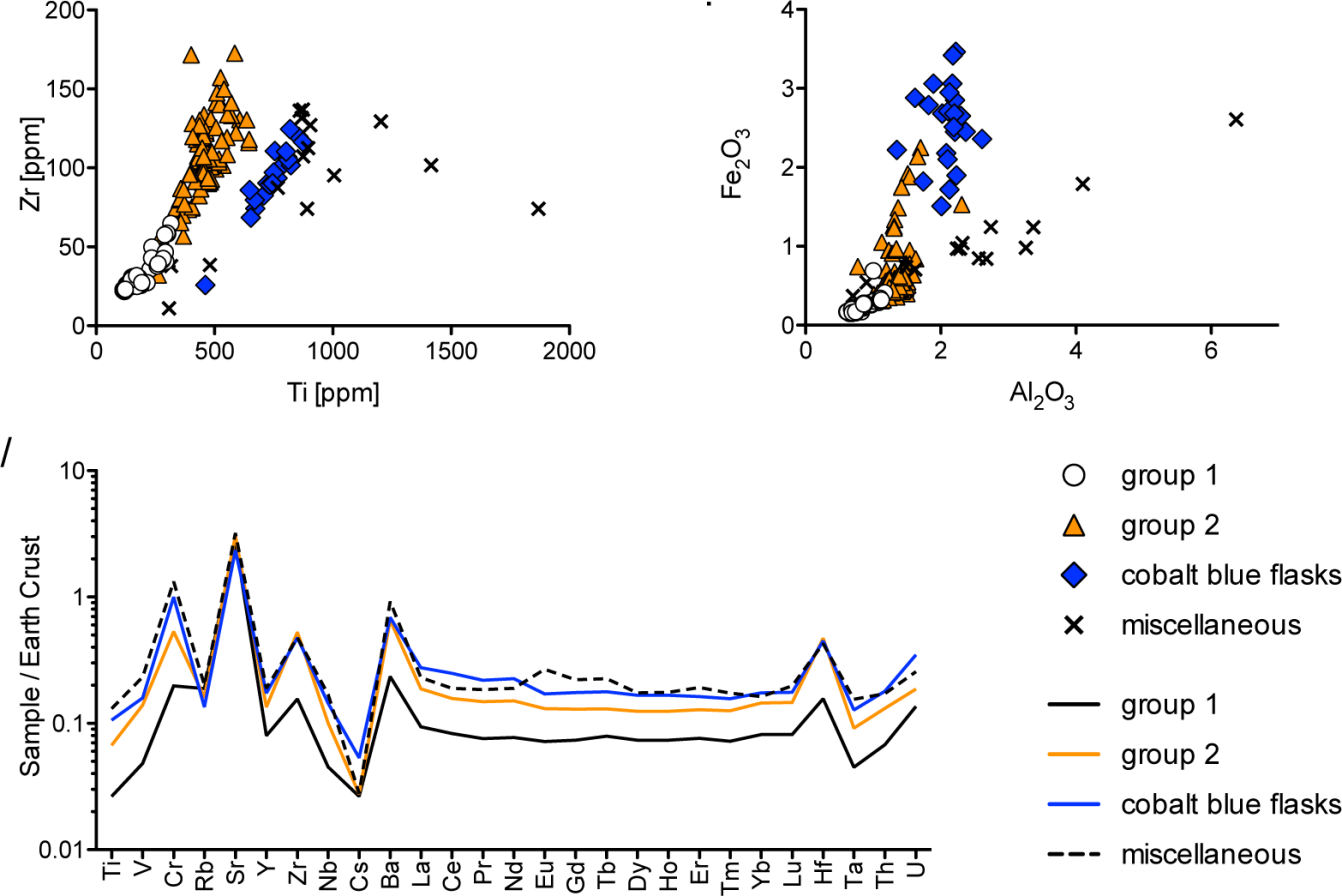
Analysis of the silica sources of the plant ash groups from Samarra. (**A**) differential zirconium and titanium correlations clearly distinguish the cobalt blue flasks from the other plant ash groups; (**B**) aluminium and iron concentrations indicate different degrees of silica related impurities; (**C**) selected trace elements, normalised to the upper continental crust compositions [36], highlighting the very low contaminants in plant ash group 1.

Plant-ash group 1 comprises almost all colourless glasses, particularly all the diamond shaped and round wall inlays (Fig 1B) and several finely decorated vessels such as a cold-painted, gilded and engraved round bottle (Berlin Sam 042) and a relief cut bowl decorated with palmette and animal motifs that is said to represent the highest standards of early Islamic glass (Berlin Sam 018) [35]. The samples of group 1 contain on average only 0.3% manganese oxide, too low to act efficiently as a decolourant. All samples have low silica-related impurities such as very low titanium and zirconium levels (Fig 5A), an average alumina content of only 0.8% (Fig 5B and Table 1) and overall very low trace elements (Fig 5C). These compositional characteristics are the result of the use of a very clean silica source (see discussion).

#### An economical variant—the use of quartz-rich sand (group 2)

Group 2 represents the bulk of the Samarra glass assemblage and includes a few colourless and aqua coloured vessels, all of the scratch-engraved samples (e.g. BM Samarra 146–153), lamps and windows, as well as all of the coloured architectural glasses such as dark purple and green drawn tesserae, chunky gold leaf tesserae and millefiori tiles (Fig 1C and S1 Table). The alumina, iron, titanium and zirconium contents as well as all other trace and rare earth elements are on average higher than in group 1 (Fig 5). This provides evidence that the silica source underlying group 2 was not as pure as the one used for group 1.

The purity of the raw materials was apparently not as decisive in the case of group 2 given the fact that colouring or de-colouring agents were added to the raw material. All the gold leaf tesserae, for example, are made from transparent glass with a greenish tinge and contain significant amounts of manganese oxide (1% - 2%) as a decolourant. The drawn tesserae are either dark green, coloured by a combination of copper and lead, or dark purple due to manganese. The millefiori tiles comprise manganese black, cobalt blue, copper-iron red, lead-tin yellow and tin white segments (S1 Table). Given that the base glass of group 2 was either coloured and/or destined for objects of lower prestige such as small ink bottles (Berlin Sam 024 MW), a more economical glassmaking recipe and technology were evidently chosen. The fact that groups 1 and 2 are compositionally closely related and of a common Mesopotamian origin demonstrates the diversity of the Abbasid glass industry and the existence of deliberate production strategies.

#### Commodity branding—the cobalt blue flasks

The 23 cobalt blue flasks form a very distinctive group (Fig 1D). The plant ash component resembles that of plant ash glass produced in Syria-Palestine or Egypt with moderate potassium and magnesium levels (Fig 2). The base glass of these bottles is derived from a silica source defined by higher titanium to zirconium ratios as well as higher alumina, trace and rare earth elements than plant ash groups 1 and 2 (Fig 5). The cobalt blue flasks exhibit also a distinct boron signature (Fig 4B). Judging from literary treatises that describe the processing of the cobalt blue pigment for glazed tiles [37], the elevated boron might in fact be the result of the addition of borax during the pre-treatment of the cobalt ore. Cobalt is strongly correlated with iron and copper, and zinc levels are likewise increased. Zinc-rich cobalt is considered diagnostic of early Islamic glass making [38–41] and was identified primarily among assemblages of supposedly Mesopotamian origin from Ctesiphon and al-Raqqa [15], Nishapur [25, 42] as well as Islamic glass beads from ninth- or tenth-century tombs in Albania [43]. However, the relative zinc levels in the cobalt blue flasks from Samarra are markedly lower than those of the Mesopotamian cobalt-zinc glasses where zinc contents typically equal or exceed the cobalt concentrations. In contrast, a cobalt to zinc ratio similar to that of the Samarra flasks (∼ 1.5x) was recently found in a set of almost identical cobalt blue bottles from Ramla dating from the ninth to the eleventh century [18] as well as in two fragments associated with the tenth-century tank furnace 2 in the glassmaking complex of Tyre [44]. Consequently, the cobalt colorant employed for the blue flasks from Samarra is more closely associated with eastern Mediterranean glassmaking, and the glasses are therefore not, as has been previously proposed, of Mesopotamian provenance [18]. The origin of the cobalt mineral itself remains elusive. The cobalt mine of Qamsar, Kāshān (Iran) that has often been cited as the source of the Islamic cobalt pigment does not appear to contain sufficient zinc to be a suitable cobalt ore [45].

According to Lamm [12], the Herzfeld excavations yielded fragments of about 170 of these elongated tubular flasks with short narrow necks and often rounded bases, made of wafer-thin cobalt blue glass (Fig 1D). The bottles themselves bear signs of mass production as the necks were crudely sawn off. Some of the necks are still closed by either cotton wool or papyrus. These types of elongated cobalt blue bottles appear to have been very widespread during the ninth to eleventh century CE. Thousands of fragments have been found all over Nishapur [19] and further examples are known from Egypt at Faiyum, Fustat and Raya, from Palestine at Tiberias, Ramla and Caesarea, from Tunisia at Sabra al-Mansuriya, as well as from the Arabian Peninsula, China and Kenya [18, 46]. The peculiar shape of these flasks is very conspicuous, which might have served the purpose of *commodity branding* to facilitate product recognition of a presumably valuable and light-sensitive content [46]. Their supply to the palaces of Samarra, where large numbers were discovered in the so-called ‘harim’ south of the throne room of the Dār al-Khilāfa [12], suggests that they served some sort of cosmetic purpose.

#### Miscellaneous vessels and imports

The miscellaneous group is as typologically diverse as it is compositionally varied. It encompasses vessels representing different decorative styles such as a relief cut vessel with animal decoration (Berlin Sam 052 MW), mould blown bowls with vertical ribs (Berlin Sam 033 MW), vessels with applied trails (Berlin Sam 054 MW), painted vessel fragments (Fig 1E), a small ink bottle (Berlin Sam 068 MW) and two marvered bowls (Berlin Sam 060 & 062 MW), a decorative technique that is commonly attributed to an Egyptian or Syrian provenance [47].

**Fig 6:**
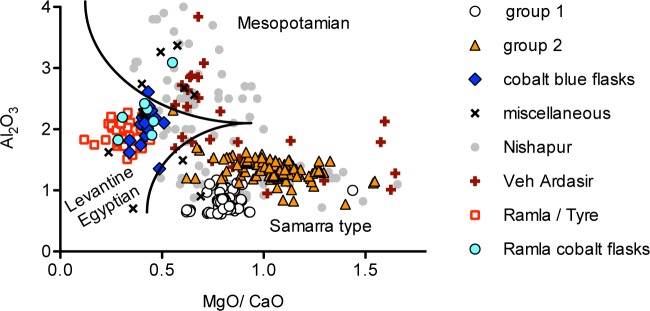
The Samarra plant ash groups compared to contemporary glass assemblages. Alumina versus magnesia to lime ratios of published data of glasses from Ramla [18], Tyre [44], Nishapur (Wypyski, in preparation) and Veh Ardasir [48, 49], indicating the proposed separation lines between early Islamic glasses from the eastern Mediterranean with low magnesia to lime ratios, the Samarra glass with high magnesia to lime ratios and low alumina levels, and Mesopotamian glasses with higher alumina concentrations typical of central Asian productions (lay-out of graph adapted from [18]).

Compositionally, the samples of this group span a wide range of impurities introduced with the silica source. The alumina levels, for example, vary from about 0.7% to over 6% (Fig 5B), while the heavy and rare earth elements are on average higher compared to the other plant ash groups from Samarra with a positive europium anomaly, probably due to the feldspathic fraction within the silica source (Fig 5C). It has to be stressed, however, that the individual samples of this group are highly variable (Fig 5A and 5B). The high impurities and variance are indicative of the use of different sands as the starting material. For instance, the samples with the highest alumina contents and low magnesium to calcium ratios (Fig 6) bear all the compositional attributes of coloured glasses from Nishapur, suggesting a central Asian glass production [18]. Given that the glasses of this group are neither compositionally nor typologically uniform, it is likely that finished glass objects arrived individually at Samarra such as, for example, a small ink bottle (Berlin Sam 068 MW) that may have been the personal possession of one of the many poets that were active during Samarra’s caliphal period. This is in contrast to the blue flasks that appear to have been imported in large numbers, implying a more centrally organised trade of these specialised objects.

## Discussion

The trace element analysis in combination with the typological affiliations supports a model whereby glass was produced at and selectively imported to Samarra. All the natron-type tesserae predate the foundation of Samarra and were imports from Syria-Palestine and/or Egypt. The compositional features of the cobalt blue flasks and some miscellaneous samples point likewise to an eastern Mediterranean provenance. Plant ash groups 1 and 2 on the other hand are undoubtedly the output of Mesopotamian glassmaking even though they represent different production strategies and raw ingredients. The analytical data proved that the silica source underlying plant ash group 1 was exceptionally pure, implying the use of quartz pebbles or a very clean quartz-rich sand. The heavy element levels (Zr, Hf, Th) of Samarra group 1 only slightly exceed those of some Iron Age plant ash glasses that are believed to have been made from quartz pebbles [50]. Nonetheless, it is near impossible to distinguish between the use of quartz pebbles and quartz rich sands at this stage. What is certain, is that the glassmakers deliberately chose a pure silica source that allowed them to closely control the ingredients and the production processes. By exploiting raw materials of high purity, the glassworkers were able to obtain truly colourless glass with no discernible tinge or aqua colour that is usually imparted to glass by the iron impurities in sand. The effort and special care that was taken to produce these colourless glasses are indicative of their cultural value and high prestige. The silica source of plant ash group 2 was richer in impurities and possibly provided a cheaper and/or less work-intensive alternative to the high-quality group 1 glasses.

Compositional features comparable to those of Samarra groups 1 and 2 have previously been attributed to Mesopotamian glassmaking as similar glasses have been found at ninth- to tenth-century Nishapur, early Islamic Ramla and Raqqa, as well as among the fourth- to fifth-century Sasanian glasses from Veh Ardašīr south of Baghdad (Fig 6) [2, 7, 15, 25, 48, 49]. Our analytical results provide the first indication of the primary production of glass at Samarra itself. The manufacture of glass in the vicinity of Samarra seems highly likely given the relative homogeneity of the bulk of the analysed assemblage (groups 1 and 2) compared, for instance, to the glass finds from Nishapur or Veh Ardašīr (Fig 6). In view of the variability of these Mesopotamian glasses, the tight clustering of Samarra group 1, the majority of which pertains to a special type of architectural glass, and its compositional resemblance to group 2 point to closely related primary productions. A regional production of glass would have ascertained the ready supply of vitreous materials for the ornamentation of the newly built palace-city of Samarra. A possible candidate of an early Islamic glass production site is al-Qadisiyya about 25 km south of Samarra, known from historical sources as the site where glass was produced (ma‘mal al-zujaj) [9]. The similarities with earlier glasses from nearby Veh Ardašīr seem to imply that the glassmakers of the early Abbasid caliphate preserved or revived and refined a centuries old Sasanian glassmaking tradition to obtain a near colourless glass for special commissions. The comparison with other Abbasid glass assemblages furthermore demonstrates a certain degree of a centralised large-scale production and interregional trade of Mesopotamian plant ash glasses during the ninth century CE.

The archaeological evidence attests to the fact that innovative wall decorations made of glass were a distinguishing and meaningful feature of Samarra’s main caliphal palace. The colourless diamond shaped and round inlays as well as the shiny dark purple and multi-coloured millefiori tiles seem to have been unique to the Dar al-Khilafa, more specifically to the audience hall of the palace and its immediate surroundings [51]. These opulent and luminous glass walls and floors would have given rise to the experience of ‘*ajab*, a sense of wonder and being in awe [52], and they may have been an allusion to Solomon’s *Glass Palace* as described in the Qur’an [53]. The lavishly decorated palaces were understood as a physical manifestation of the ideal ruler [14]. It may thus not come as a surprise that no expenses were spared and special care was taken in the manufacture of the decorative architectural glasses of group 1 the aesthetic qualities of which were evidently regarded as ideologically important.

The possible existence of primary glassmaking and the diversity of architectural glasses at Samarra during the Abbasid period invites some tantalising questions about the import of mosaic tesserae. It is a widely held assumption that wall mosaics were intrinsically Byzantine, to the effect that in the early eighth century the Byzantine emperor allegedly supplied the materials and workforce for the mosaic decoration of the great Umayyad mosques in Damascus and Medina [54]. Support for this claim is found in numerous Arab sources, most notably in the *Ta’rikh al-Rusul wa’l-Muluk* (History of Prophets and Kings) of al-Tabari (838–923 CE), according to which the Sahib al-Rum (the Byzantine emperor) sent money, glass tesserae and workmen at the request of the Caliph al-Walid for the decoration of the Great Mosque in Medina [55, 56]. Modern scholarship is divided over whether or not the account contains any truth, not least because al-Tabari’s chronicle was written more than two centuries after the event. There can be no doubt, however, that textual sources contain valuable records of the construction of cultural identity at the time when they were written. Al-Tabari wrote his great chronicle during the latter part of the ninth century, and thus in living memory of the foundation of Samarra. Our analytical data provide unequivocal evidence that the caliphs who extended and embellished the new Abbasid capital indeed imported mosaic tesserae. This study thus furnishes material proof that al-Tabari’s account has a historical basis, even though the anecdote might not be factually accurate. Nonetheless, the document must be considered authentic in relation to the symbolic significance of glass in ninth-century Samarra. It sheds light on architectural and artistic practices as well as international relations.

## Conclusion

Our high-resolution trace element analyses of a statistically significant number of samples from a well-dated single site of exceptional archaeological relevance provide the first clear evidence for the organisation of the Abbasid glass industry and trade as a whole. The glass finds from Samarra demonstrate the specific commission of vitreous materials for the newly built palace city, concurrent with the interregional exchange of specialised objects. Variations in the mineralogical characteristics of the raw ingredients revealed different technological choices, identifying the possible use of quartz pebbles for the manufacture of a particular type of colourless architectural glass. The implementation of this labour-intensive production technology points to a considerable degree of sophistication and by extension aesthetic and cultural value attributed to vitreous materials during the ninth century. The similarity between the two types of the architectural glasses that make up the bulk of the assemblage (75% of the analysed samples) is indicative, we believe, of a local production in the vicinity of Samarra and a centrally organised system of production and supply. Due to the robust sampling strategy and statistically valid approach, our study establishes the extent of variability within a single assemblage and thus provides an essential tool to categorise and provenance early Islamic plant ash glasses more generally. By integrating trace element data with archaeological, art historical and literary evidence, this study exemplifies the potential of chemical analysis of ancient glass to elucidate fundamental historical phenomena in relation to technological innovations and wider cultural and intellectual developments.

## Supporting information

S1 Table LA-ICP-MS data of the ninth-century glass from Samarra (Iraq). (XLSX)

S2 Table LA-ICP-MS data of glass standards in comparison with published values. (PDF)
