# Supplementary material for: The glass walls of Samarra (Iraq): Ninth-century Abbasid glass production and imports
Source: PLoS One. 2018 Aug 22;13(8):e0201749. doi: 10.1371/journal.pone.0201749 (PMC6104971; doi:10.1371/journal.pone.0201749)
Supplement: S2 Table — (PDF) [file pone.0201749.s002.pdf]

S2 Table: LA-ICP-MS data of glass standards in comparison with published values

	Li_2O	B_2O_3	Na_2O	MgO	Al_2O_3	SiO_2	P_2O_5	Cl	K_2O	CaO	TiO_2	V_2O_5	MnO	Fe_2O_3	CoO	NiO	CuO	Rb_2O	SrO	ZrO_2	SnO_2	Sb_2O_3	BaO	PbO	Bi
Corning A (n=63)	0.01	0.21	13.84	2.51	0.91	66.87	0.12	0.13	2.85	5.78	0.78	0.01	1.02	1.10	0.17	0.02	1.16	0.01	0.10	0.01	0.17	1.62	0.45	0.06	0.00
relative σ	5.94	3.49	1.29	2.52	3.04	0.47	11.93	11.42	1.75	2.07	2.23	1.96	2.30	3.18	2.29	3.10	2.52	1.64	1.60	4.01	1.84	1.71	2.41	4.83	2.75
Vicenzi[1]	0.01	0.20	14.30	2.66	1.00	66.56	0.13	0.10	2.87	5.03	0.79	0.01	1.00	1.09	0.17	0.02	1.17	0.01	0.10	0.01	0.19	1.58	0.56	0.073[2]	0.001
accuracy	3.87	3.66	3.23	5.83	8.56	0.47	9.69	34.71	0.58	14.97	1.54	5.11	1.68	1.31	0.33	12.92	1.25	5.54	2.67	7.22	12.07	2.65	19.13	12.54	14.70
NIST612 (n=59)			13.54		2.08	72.53				11.54															
relative σ			1.16		3.12	0.43				1.69															
Jochum[3]			14.00		2.06	71.70				11.93															
accuracy			3.28		0.96	1.15				3.30															

	V	Cr	Mn	Co	Ni	Cu	Zn	Ga	As	Rb	Sr	Y	Zr	Nb	Mo	In	Sn	Sb	Cs	Ba	La	Ce	Pr	Nd	Sm	Eu	Gd	Tb	Dy	Ho	Er	Tm	Yb	Lu	Hf	Ta	Pb	Bi	U
NIST612 (n=69)	37.51	35.76	40.26	35.55	37.62	36.65	36.61	36.53	34.45	31.88	75.82	37.40	37.44	34.83	32.84	37.33	34.25	35.90	41.17	36.28	37.92	38.36	36.97	35.26	37.11	36.26	34.68	37.30	34.31	37.95	35.26	35.67	38.64	36.77	35.20	31.27	32.48	30.02	37.61
relative σ	1.99	8.73	2.47	2.77	3.14	3.90	3.04	2.18	4.38	1.54	2.22	1.37	1.89	2.36	2.46	2.06	2.13	6.65	1.69	1.55	4.21	4.44	2.67	2.03	2.21	1.46	5.91	1.48	1.51	1.64	2.16	1.87	2.47	2.05	1.71	1.39	11.27	2.64	3.06
Jochum[3]	39.90	36.26	39.40	34.82	37.90	38.70	41.20	36.90	36.80	31.07	78.51	40.14	40.36	41.54	35.79	38.90	40.90	36.40	42.14	39.37	34.65	37.25	37.31	34.96	37.15	35.60	38.56	39.96	36.15	38.69	38.86	36.80	39.84	37.00	37.13	38.17	38.60	30.20	37.68
accuracy	5.98	1.37	2.19	2.09	0.74	5.29	11.13	1.00	6.40	2.60	3.42	6.83	7.22	16.16	8.25	4.05	16.27	1.37	2.30	7.86	9.44	2.99	0.91	0.87	0.12	1.86	10.05	6.66	5.09	1.91	9.26	3.07	3.00	0.62	5.19	18.08	15.85	0.60	0.20

1. Vicenzi EP, Eggins S, Logan A, Wysoczanski R. Microbeam characterization of corning archeological reference glasses: new additions to the smithsonian microbeam standard collection. Journal of Research of the National Institute of Standards and technology. 2002;107(6):719.
2. Wagner B, Nowak A, Bulska E, Hametner K, Günther D. Critical assessment of the elemental composition of Corning archeological reference glasses by LA-ICP-MS. Analytical and bioanalytical chemistry. 2012;402(4):1667-1677.
3. Jochum KP, Weis U, Stoll B, Kuzmin D, Yang Q, Raczek I, et al. Determination of reference values for NIST SRM 610–617 glasses following ISO guidelines. Geostandards and Geoanalytical Research. 2011;35(4):397-429.
